# Supplementary material for: “I feel like I’m in a revolving door, and COVID has made it spin a lot faster”: The impact of the COVID-19 pandemic on youth experiencing homelessness in Toronto, Canada
Source: PLoS One. 2022 Aug 22;17(8):e0273502. doi: 10.1371/journal.pone.0273502 (PMC9394800; doi:10.1371/journal.pone.0273502)
Supplement: Attachment — Submitted filename: Response to Reviewers.docx [file pone.0273502.s001.docx]

PONE-D-22-01338

“I feel like I’m in a revolving door, and COVID has made it spin a lot faster”: The impact of the COVID-19 pandemic on youth experiencing homelessness in Toronto, Canada – Response to reviewers

Thank you to the editor and reviewers for your thoughtful comments on our manuscript. We address each of the comments in greater detail below. If there are any other comments or points that arise during the review process, please let us know.

Journal Requirements:

1. Please ensure that your manuscript meets PLOS ONE's style requirements, including those for file naming.
   a. The manuscript has been revised to conform to PLOS ONE’s style requirements, and the file names have been updated for this resubmission. Apologies for this mistake.
2. We note that you have provided funding information. However, funding information should not appear in the Funding section or other areas of your manuscript. We will only publish funding information present in the Funding Statement section of the online submission form. Please remove any funding-related text from the manuscript and let us know how you would like to update your Funding Statement.
   a. All funding-related text has been removed from the manuscript. The funding statement provided in the online submission form is accurate and up-to-date, and does not need to be updated.
3. In your Data Availability statement, you have not specified where the minimal data set underlying the results described in your manuscript can be found.
   a. We have modified our data availability statement and added the University of Toronto’s Social Science Ethics Review Board as the contact for anonymized data. The revised statement is as follows:

“Data cannot be shared publicly as participants did not consent to the release of transcripts in a public domain. In addition, interview data holds sensitive information from youth experiencing homelessness and the staff who serve them in Toronto, Ontario. These populations are small, and thus publicly sharing data would compromise the level of risk of participating in the research—particularly given the marginalization that youth experiencing homelessness already experience and the ways in which staff discussed their places of employment. Researchers who wish to access anonymized interview data are encouraged to contact the University of Toronto’s Social Science Research Ethics Board to request access at ethics.review@utoronto.ca, +1 (416) 946-3273.”

Additional Editor Comments:

1. The manuscript is too long, try to shorten it.
   a. We have substantially revised the manuscript to reduce the length. As a result, we have reduced the word count of the body text from 8641 words to 6710.
2. Use continuous line numbers (do not restart the numbering on each page).
   a. Apologies for this. Continuous line numbers have been added to the manuscript.
3. Table 1 and Table 2 are results, not methods. So, remove them from methodology section and add them to results section.
   a. Thank you for pointing out this mistake. Tables 1 and 2 have been moved to the results section.
