# Supplementary material for: “I feel like I’m in a revolving door, and COVID has made it spin a lot faster”: The impact of the COVID-19 pandemic on youth experiencing homelessness in Toronto, Canada
Source: PLoS One. 2022 Aug 22;17(8):e0273502. doi: 10.1371/journal.pone.0273502 (PMC9394800; doi:10.1371/journal.pone.0273502)
Supplement: Attachment — Submitted filename: Response to Reviewers.docx [file pone.0273502.s002.docx]

PONE-D-22-01338R1

“I feel like I’m in a revolving door, and COVID has made it spin a lot faster”: The impact of the COVID-19 pandemic on youth experiencing homelessness in Toronto, Canada – Response to reviewers

We are very grateful to the editor and reviewers for your thoughtful comments on our manuscript. We address each of the comments in greater detail below. If there are any other comments or points that arise during the review process, please let us know.

Reviewer #1’s comments:

1. It is suggested to explain an ethical section where the approval of the ethics committee and the verbal informed consent given by the participants are mentioned, given the vulnerability of the study population.
   a. Thank you for this comment. We have added a subsection on research ethics to the methodology section of the manuscript where we detail the study’s ethics approval and the verbal informed consent process.
2. When the case of 2SLGBTIQ+ youth is presented, it is recommended to have first-person quotes from their experience, not only focused on the discourse of the shelter workers.
   a. This is a very important point raised by both reviewers. The section on 2SLGBTQ+ youth’s experiences has been updated to include quotes from 2SLGBTQ+ youth themselves, who describe experiences of isolation and difficulty accessing services.
3. It is suggested to share the limitations of the study in the discussion section if they exist.
   a. The manuscript has been updated to include a section on limitations alongside the discussion. This section addresses the study’s limitations and suggests potential areas for future research.
4. It would be interesting to include a reflection from the gender perspective on the women experience who participated less than man in the study.
   a. Thank you very much for this comment. While an analysis of the gendered impacts of the pandemic for youth experiencing homelessness is important, it is unfortunately a limitation of this study given the interview protocol and sample. We have added this as a limitation in the limitations section of the manuscript and call for future research to address this gap.
5. In the final list of references the year of publication corresponds to the APA style, it is needed to change the references to Vancouver format.
   a. The reference list has been updated to be consistent with the Vancouver citation style guide.

Reviewer #2’s comments:

1. Regarding the sections where the experience of subpopulations is developed, the one that addresses the 2SLGBTQ+, lacks the perspective of the 2SLGBTQ+ population interviewed, as only staff quotes were incorporated.
   a. Thank you for this very important comment. As mentioned above, we have added quotations from 2SLGBTQ+ youth to this section, where they share first-hand their experiences of isolation and difficulty accessing supports.
2. Also, according to the consolidated criteria for reporting qualitative research, an identification should be added to each quotation, for example, a participant number (protecting the anonymity of the participants).
   a. The manuscript has been revised to include unique participant codes for each quotation. Quotes from youth are prefixed by ‘Y’, and quotes from staff are prefixed by ‘ST’. This coding formula is described in the methods section of the manuscript.
3. Along the report, some minor language editing is required, for example, with repeated words like "support" and "impacts".
   a. Thank you for catching this. The manuscript has been revised to minimize repeated words.
4. Finally, a few format aspects should be addressed, like the size of the numbers in the references and the incorporation of page numbers.
   a. The reference list has been edited for consistency in font size.
